# Social Engagement in HIV Cure (Research) in the Netherlands: Understanding the Perceived Necessity and Concerns of People With HIV

**DOI:** 10.1097/QAI.0000000000003429

**Published:** 2024-06-21

**Authors:** Maaike A. J. Noorman, John B.F. de Wit, Tamika A. Marcos, Sarah E. Stutterheim, Kai J. Jonas, Chantal Den Daas

**Affiliations:** a Department of Interdisciplinary Social Science, Utrecht University, Utrecht, the Netherlands;; b Department of Work and Social Psychology, Maastricht University, Maastricht, the Netherlands;; c Department of Health Promotion and Care and Public Health Research Institute, Maastricht University, Maastricht, the Netherlands; and; d Health Psychology Group Institute of Applied Health Sciences, University of Aberdeen, Aberdeen, United Kingdom.

**Keywords:** HIV, HIV cure, people with HIV, social engagement, MIPA

## Abstract

**Objective:**

As the developments toward HIV cure are gaining prominence, engagement of people with HIV (PWH) becomes crucial. The aim of this study was to gain deeper insight into how PWH engage with HIV cure (research) by exploring their perspectives regarding awareness, importance, and meaning of HIV cure (research).

**Methods:**

Semistructured interviews were conducted with 30 purposively sampled PWH in the Netherlands. Interviews were transcribed verbatim, and thematic data analysis was undertaken.

**Results:**

The overall necessity for an HIV cure was high, but personal perceived necessity varied based on participants' lived experiences with HIV. Most participants saw less personal necessity due to their high quality of life, while those more affected by their HIV status expressed a stronger need for a cure. Participants held high expectations for an HIV cure, considering viral eradication as the only cure. Lack of awareness of potential HIV cure strategies and techniques shaped concerns. Antiretroviral treatment-free viral suppression was met with concerns and not considered a cure, but rather an improved form of treatment. Engagement with HIV cure manifested in various forms and levels, with only a few participants actively engaged or willing to participate in HIV cure clinical research. Most participants were interested but inactive and unsure about participating in HIV cure clinical research.

**Conclusions:**

PWH's social engagement in HIV cure (research) is shaped by their perceived necessity and concerns. Understanding these perceptions can increase social engagement.

## Abstract 2

Supplemental Digital Content is Available in the Text.

## INTRODUCTION

Since the 1980s, HIV treatment has come a long way, transforming HIV into a manageable chronic condition.^1^ To date, 5 individuals have been cured of HIV following stem cell transplantation.^2–6^ However, this approach is highly invasive and not universally applicable to all people with HIV (PWH). Consequently, efforts now focus on developing a less invasive cure that can either eliminate HIV (ie, HIV eradication) or achieve antiretroviral treatment (ART)-free viral suppression (ie, functional cure, HIV remission, or ART-free viral suppression).^7,8^ Recognizing the importance of an HIV cure, the International AIDS Society established a Global Scientific Strategy,^7^ which, in addition to basic and clinical research, highlights the significance of social engagement research, exploring the views and experiences of, and support for, affected communities in relation to HIV cure and HIV cure research.

The importance of social engagement research has been demonstrated by HIV prevention and treatment research^9^ as it not only facilitates the translation of basic science but also promotes social justice by empowering PWH to influence research practices and make informed decisions affecting their health. The increasing number of publications on social engagement research in HIV cure (research) also reflects an increase in attention to this topic in HIV cure research.^10^ While most studies focused on perspectives on HIV cure research, including perceptions on clinical trial participation, and facilitators and barriers of HIV cure research, fewer have explored perspectives on HIV cure itself, and its meaning and impact for PWH. To enhance our understanding of PWH's engagement, greater consideration should be given to perspectives on HIV cure and, where feasible, efforts should be made to integrate these with perceptions of HIV cure research to better understand PWH's decision-making processes about social engagement in HIV cure (research).

The aim of this study was to improve our understanding of PWH in the Netherlands' social engagement with HIV cure and HIV cure research by exploring their awareness, perceived importance, and ascribed meaning of HIV cure (research). As such, this study explored these concepts through the lens of the Necessity-Concerns Framework (NCF),^11,12^ which has previously been applied to HIV treatment research.^13,14^ NCF distinguishes 2 types of considerations that may influence the engagement of PWH with HIV cure (research): the perceived necessity of an HIV cure and the perceived concerns regarding an HIV cure. Necessity beliefs refer to the extent to which PWH view the perceived benefits of an HIV cure as critical, while concern beliefs encompass their assessment of the potential adverse impacts of an HIV cure.^11^

## METHODS

### Study Context and Design

This qualitative study was part of the Dutch NL4cure initiative, aimed at advancing HIV cure research through a collaboration of diverse stakeholders.^15^ The Netherlands achieved the Joint United Nations Programme on HIV/AIDS (UNAIDS) 90-90-90 targets, with approximately 97% of PWH receiving ART achieving viral suppression.^16^ However, despite ART's effectiveness, it is not a cure. Research indicates that PWH on ART in the Netherlands still experience side effects, challenges in disclosing their HIV status, social stigma, and self-stigma, highlighting the need for research on HIV cure options and their associated impacts.^17^

This study took an explorative approach to study the perspectives of PWH in the Netherlands on awareness, importance, and meaning of HIV cure (research). Community and professional advisory boards played an essential role in guiding the study, contributing to its design, interview guide development, recruitment, and interpretation of preliminary findings. The community advisory board included 5 PWH and 1 cisgender man without HIV. The professional advisory board comprised 6 professionals, including 2 HIV clinicians, 2 HIV specialized nurses, 1 social scientist, and a board member of the Hiv Vereniging (Netherlands Association of People with HIV). The study protocol was approved by the Faculty Ethics Review Board of the Faculty of Social and Behavioral Science, Utrecht University (#20-373), and by the Ethics Review Committee Psychology and Neuroscience, Maastricht University (188 11 02 2018_S21).

### Participants and Recruitment

We employed purposive sampling to select eligible participants, who were adults (>18 years) with HIV living in the Netherlands. The goal was to ensure diversity in terms of gender, age, sexual orientation, migration background, and years since diagnosis. Our recruitment strategy involved a post on the Hiv Vereniging website, which was also shared across various social media platforms through virtual snowball sampling. In addition, an HIV specialist nurse assisted with recruitment. When necessary, we tailored the original post to recruit individuals with specific characteristics. Interested individuals contacted the researchers via email or phone and received a participant information sheet explaining study objectives, the right to withdraw at any time, data storage and access, and confidentiality measures.

### Procedures

Participants who agreed to participate were scheduled for interviews. Interviews were conducted in both Dutch and English. Because of COVID-19 restrictions, interviews were conducted online using Microsoft Teams or by telephone. Before the start of the interview, participants had the opportunity to ask questions about the study and provided online and verbal informed consent. Participants received a reimbursement of €40.

### Data Collection

Between April 2021 and February 2022, 2 researchers (M.A.J.N. and T.A.M.) conducted 30 semistructured interviews. Participants self-reported their sociodemographic information and HIV-related characteristics through an online questionnaire before the interviews. The interviews had a conversational style with a flexible, topic-oriented structure outlined in an interview guide (see the Supplemental Digital Content, http://links.lww.com/QAI/C281). The interview guide included open-ended questions and follow-up prompts about participants' current experiences with HIV, awareness, importance, and meaning of HIV cure (research), stigma in relation to HIV cure, views on meaningful involvement of PWH, and communication strategies in HIV cure research. We report only the results related to awareness, importance, and meaning here, while the remaining findings will be reported elsewhere. Interviews were audio recorded and ranged from 45 to 103 minutes (M = 72 minutes). Data collection continued until saturation, indicated by the emergence of no new themes and the richness and complexity of perspectives.^18^ The interviewers initially established saturation through discussion and then conferred with the research team to determine if the data collection process could be finalized.

### Data Processing and Analysis

Audio recordings were transcribed verbatim, and transcripts were reviewed by 2 researchers (M.A.J.N. and T.A.M.) for completeness and accuracy. Data analysis was guided by thematic analysis outlined by Braun and Clarke.^19^ Thematic analysis, a flexible and iterative process, enabled us to uncover underlying ideas, assumptions, and conceptualizations of participants.^18,19^ NVivo20 was used to inductively generate initial codes as transcripts were read by M.A.J.N. Similar codes were grouped together to create a comprehensive codebook. Coding reliability was evaluated by T.A.M. in one-third of the interviews (n = 10) and no new codes emerged. The codebook was subsequently used to identify preliminary themes, which were refined through discussions within the research group until clear and distinct themes were captured.^19^ Data were analyzed in their original language. To enhance clarity and conciseness in writing, Grammarly and ChatGPT were used.

## RESULTS

### Participant Characteristics

Thirty PWH, aged 27–78 years, averaging 49.5 years, were recruited. Nineteen participants identified as cisgender men, 10 as cisgender women, and 1 as transgender woman. Seventeen participants identified as gay, 10 as straight, and 3 as bisexual. Seven participants had a migration background from countries, including Nigeria (n = 2), Cuba, Curacao, Germany, Romania, and Zimbabwe (1 each). Sixteen participants had a steady partner (ie, spouse, romantic partner, open relationship). The sample included 13 people who have been living with HIV before the advent of combination of ART in 1996, 14 participants who were diagnosed between 1997 and 2019, and 3 participants who were recently diagnosed (ie, in the year before the interview). All participants were taking ART, with all but one having an undetectable viral load.

### Identified Themes

We identified 3 overarching themes: (1) the perceived necessity of an HIV cure, (2) HIV cure concerns and expectations, and (3) willingness to engage with and participate in HIV cure research (Table 1).

**TABLE 1.** Identified Themes, Key Findings, and Implications

Theme	Key Findings	Implications
The perceived necessity of an HIV cure	•The overall perceived necessity for an HIV cure is high •The personal perceived necessity depends on participants' personal experiences with HIV	Clinicians and researchers should recognize the diverse experiences of PWH in the context of HIV cure research to ensure that the development of an HIV cure aligns with their needs and lives
HIV cure expectations and concerns	•HIV cure expectations depend on the personal perceived necessity •A lack of awareness of potential HIV cure strategies causes concerns	To decrease concerns, manage expectations, and increase acceptability and engagement among PWH, clinicians, and researchers should prioritize early nonscientific dissemination of potential HIV cure strategies, along with the accompanying research procedures and risks
Willingness to engage with and participate in HIV cure (research)	•Willingness to engage with and participate in HIV cure (research) is determined by participants' perceived necessity and concern •Engagement can be active and passive, or people are skeptical •Participants are willing, unwilling, or uncertain about participation in clinical trials	Clinicians and researchers should take PWH's perceived (personal) necessity and their perceived concerns into account when trying to engage or recruit them

### The Perceived Necessity of an HIV Cure

All participants perceived the development of an HIV cure as highly necessary, given their perception of HIV as a serious and highly stigmatized chronic condition. Most believed that a cure was especially important for those without access to ART or in environments marked by pervasive HIV-related stigma. As illustrated by a straight cisgender woman in her 60s diagnosed with HIV between 1997 and 2019, an HIV cure is “More for lower-income countries […] where HIV is just denied or sometimes difficult to treat.” Furthermore, participants emphasized the cure's significance for future generations and those newly diagnosed with HIV, and its potential societal benefits, such as the reduction in healthcare costs by eliminating the need for lifelong HIV treatment.

While all participants perceived the development of an HIV cure as highly necessary, the perceived personal necessity varied and depended on their individual experiences of living with HIV. Most participants expressed that they did not personally deem an HIV cure to be essential. They had learned to effectively manage their lives with HIV due to positive experiences with ART. Nonetheless, most participants still desired a cure as they continued to rely on ART, attended clinic appointments, and encountered challenges related to travel and obtaining insurances. As exemplified by a gay cisgender man in his 60s diagnosed with HIV before 1997: “I believe very little would change, especially since my medication is working so well, and I do not experience any side effects […] but in one way or another [with a cure], you would no longer have any more inconveniences.”

The desire for an HIV cure was stronger among participants who felt more impacted by their HIV status, particularly those who concealed it due to fear of judgment. They believed that a cure could help them form better connections and alleviate dating challenges, especially for heterosexual and bisexual participants. A straight cisgender man in his 40s diagnosed with HIV between 1997 and 2019, described: “Before you have sex for the first time, […] ladies are frightened because no one knows about it [HIV].” Participants facing health challenges also expressed a stronger need for an HIV cure, believing it could improve their overall well-being, alleviate chronic fatigue, boost their immune system, and address long-term health concerns, including aging-related issues, susceptibility to other illnesses, and ART resistance concerns.

Some participants stated not needing nor desiring an HIV cure. Those who had experienced irreversible side effects from HIV or ART believed that a cure would not fix these, as asked rhetorically by a straight cisgender woman in her 60s diagnosed with HIV between 1997 and 2019: “Would it matter much for me? […] I do not get my eyesight back.” Several other participants feared that a cure might lead to a loss of their HIV-positive identity, community connection, or even the sexual satisfaction they currently experienced.

### HIV Cure Expectations and Concerns

Participants held high expectations for an HIV cure, envisioning it as a noninvasive, quick, side effect–free, and universally accessible intervention that eliminates HIV from the body. However, most participants recognized that this ideal cure is not (yet) realistic, given HIV's adaptability and ability to hide within the body. Furthermore, most participants were familiar with Timothy Ray Brown, the first person cured of HIV through highly invasive stem cell transplantation. Several participants explicitly expressed their reluctance to pursue a cure through such a method, as exemplified by a straight cisgender woman in her 50s diagnosed with HIV before 1997. After meeting him, she stated: “I would not like to trade places with him. I do not want to get very sick.”

What a realistic cure would entail and how it would be developed was difficult to imagine for most participants. Their knowledge of potential HIV cure strategies was limited. Only a few participants were aware of specific strategies and techniques such as clustered regularly interspaced short palindromic repeats (CRISPR)-Cas (CRISPR-associated proteins), kick and kill, and block and lock. However, even fewer felt they understood these strategies or techniques, which participants considered highly medical. A cisgender woman in her 40s diagnosed with HIV before 1997 stated: “Crisscross [CRISPR-Cas] is a new technique that could get HIV under control, but that is so technical. So, I do not know more than this.”

Participants were critical of the development of a practical HIV cure, preferring strategies aiming to remove HIV from the body over ART-free viral suppression. ART-free viral suppression of HIV raised concerns, particularly regarding the possibility of viral rebound after treatment cessation, which most participants found unacceptable, not only for their own health but also because of transmission risks associated with this. Hence, most participants did not consider ART-free viral suppression a cure. Some even preferred ART, citing a desire for more control, as explained by a gay cisgender man in his 30s diagnosed with HIV between 1997 and 2019: “I would rather know where I stand. That is then either take a pill or you are done with it.” However, for many, ART-free viral suppression was seen as an improved treatment option, like long-acting injectables. A gay cisgender man in his 40s with a migration background diagnosed with HIV between 1997 and 2019 expressed: “Every step is one in the right direction. If you can use less medication, it is good.”

### Willingness to Engage With and Participate in HIV Cure (Research)

For most participants, engagement with HIV cure (research) was passive. They typically stayed informed through news articles or discussions with their HIV nurses. Only a minority, often those with a stronger need for a cure, took a proactive approach, seeking out information, attending community events, or participating in advisory boards. Some also expressed disinterest as they had become skeptical over the years or had grown weary of the topic.

Many participants hesitated to engage in HIV cure research through participation in clinical trials as it was difficult to envision what participation would entail and what the associated risks were. Several participants described that they would need more information on the exact risks. Perceived risks included concerns about potential side effects, and worries about viral load increase and the body's response to it, potential ART resistance, and the transmission of HIV during analytical treatment interruptions. As a straight cisgender man in his 40s diagnosed with HIV between 1997 and 2019 described: “I really want to participate in research, […], but if, for example, I were to stop taking my pills […] I do not know if I would do that […] because I can infect someone else.” A few participants, often driven by a strong belief in science and altruistic motives, expressed a willingness to participate in HIV cure clinical trials. For instance, a gay cisgender man in his 30s diagnosed with HIV between 1997 and 2019 said: “I am here because of others because they once started AZT for me.” Conversely, some participants were unwilling to participate, as they did not want to risk their current quality of life for uncertainty. A straight cisgender woman in her 40s diagnosed with HIV before 1997 said: “Research suggests uncertainty. Thus, as long as I am not with my back against the wall I would not participate in research.”

## DISCUSSION

The aim of this study was to better understand the social engagement of PWH in the Netherlands with HIV cure (research) by assessing their awareness, perceived importance, and ascribed meaning of HIV cure (research). We employed the NCF as a theoretical lens, which offers a valuable framework for examining the intricate interplay between individuals' necessity and concerns beliefs. Following the NCF, our results show that the interplay between participants' perceived necessity for an HIV cure, shaped by their experiences of living with HIV, and their concerns, influenced by their HIV cure treatment expectations and understanding, shape participants' engagement with HIV cure (research).

While all participants recognized the overall importance of an HIV cure, the personal necessity varied based on individual experiences of living with HIV. In our Dutch sample, many PWH had a lower perceived personal necessity due to their good quality of life. Nevertheless, the desire for an HIV cure remained significant. PWH who were more profoundly affected by their HIV status expressed a stronger need for a cure. In addition, a small subset of participants reported neither needing nor desiring a cure, believing it would not significantly improve their lives. These diverse perspectives align with a systematic review where responses to HIV cure development were categorized into neutral, positive, and negative.^10^ Future quantitative research could delve deeper into the factors influencing these different perspectives on HIV cure (ie, age, gender, sexual orientation, years since diagnosis). Recognizing these diverse experiences of living with HIV within the context of HIV cure research is crucial, as it contributes to understanding diverse groups in HIV cure research and could facilitate broader social engagement.^7^

In our study, participants also expressed concerns about a potential HIV cure based on their understanding of an HIV cure. Like prior research,^17,20–23^ most participants only considered complete virus eradication as a cure. Interestingly, they were aware of some of the challenges involved in achieving such a cure, as all participants displayed substantial knowledge about the complexity of HIV and the invasiveness of stem cell transplantation. This awareness might explain their positive view of ART-free viral suppression as an enhanced treatment option, resembling long-acting ART. Like other studies,^17,20–23^ some concerns about ART-free viral suppression were reflected in our findings, as participants acknowledged the potential for HIV to resurface while it remains in the body. Nonetheless, most participants believed that ART-free viral suppression represented a positive progression toward the ultimate cure: HIV eradication. Our findings, akin to earlier studies on preexposure prophylaxis uptake,^24–26^ underscore the importance of creating awareness regarding HIV cure developments, which may increase the acceptability and eventual uptake of a potential HIV cure, such as ART-free viral suppression.^27^

Furthermore, participants had concerns about the intensity and side effects of a potential cure. However, their ability to define these concerns was hindered by a lack of specific knowledge about cure strategies and research procedures. To further enhance HIV cure (research) understanding, we recommend that researchers prioritize the nonscientific dissemination of potential HIV cure strategies, techniques, and their associated risks.^27^ Following good participatory practice by actively exchanging information early on,^28^ researchers could increase awareness and knowledge among PWH and provide opportunities for PWH to develop informed perspectives on potential HIV cure strategies. This, in turn, empowers them to make more informed decisions about the acceptability of these strategies and whether they would be interested in participating in related research endeavors.^27,28^

Beyond clinical research participation, our findings also demonstrated different forms of engagement with HIV cure research, including staying informed, attending community events, or engaging with scientists through community advisory boards. Moreover, we found different levels of engagement. Through the NCF as a theoretical lens,^11^ we can explain the different levels of engagement in this study. High general perceived necessity and lower personal perceived necessity led to passive interest, while higher personal perceived necessity was associated with more active engagement. Moreover, we can attribute the uncertainty most participants felt toward participation in HIV cure clinical trials to a perceived lower personal importance and uncertainty of associated risks. The few willing to participate in clinical trials were driven by high perceived general necessity and sense of altruism, coupled with minimal perceived concerns, likely influenced by their trust in the medical community. These facilitators were also reflected by earlier research.^10^ Conversely, the few unwilling to participate in clinical trials expressed a low personal perceived necessity, as they were already living well with ART and not willing to jeopardize their current standard of living for perceived concerns associated with clinical trial participation. These sentiments have also been reflected by earlier studies.^29,30^

The uncertainty toward clinical trial participation that most participants in our study felt contrasted with the results of a systematic review, which showed an overall high willingness to participate in clinical trials.^10^ However, they align with several qualitative studies,^29,31–33^ supporting arguments that hypothetical willingness in quantitative studies may have been overestimated.^34,35^ Given that qualitative research is participatory and offers opportunities for meaningful engagement,^36^ an explanation for these different observations could be that participants during qualitative studies have an opportunity to develop a deeper understanding of HIV cure trials,^32^ allowing them to evaluate the risks associated with different strategies such as analytical treatment interruption or cell and gene therapy.^29,31^ Therefore, we recommend further research focused on understanding how PWH perceive and comprehend HIV cure (research), as this understanding seems to shape their perceived concerns, which significantly influences their decision to engage with and participate in HIV cure research.

While our study significantly contributes by introducing the NCF as a theoretical framework for understanding social engagement in HIV cure research, it has limitations. Although highly diverse in terms of gender, age, sexual orientation, migration background, and years since diagnosis, all participants were from the Netherlands with access to ART. This context-specific aspect may potentially restrict the applicability of our findings to regions where universal ART is not available. In addition, it is worth noting that the topic of HIV cure (research) can be quite abstract and complex as we asked participants about something that does not yet exist. The reality of an HIV cure may differ from our findings. Moreover, it is important to acknowledge that the interpretation of qualitative data can be subjected to researcher biases. To enhance the reliability of our findings, we implemented a triangulation approach. This involved having 2 independent researchers conduct and analyze the interviews, alongside extensive discussions within the research team, community advisory board, and professional advisory board to address potential researcher biases.

## CONCLUSION

Our study revealed that PWH's engagement with HIV cure (research) was, on the one hand, tied to their perceived necessity, influenced by their experience of living with HIV. On the other hand, it was determined by perceived concerns, linked to their awareness of HIV cure developments. By understanding the perceived necessity for an HIV cure, it becomes possible to align HIV cure (research) with specific needs and expectations. Increasing awareness can address perceived concerns providing PWH the opportunity to develop informed perspectives about meaningful engagement with or participation in HIV cure research. This will ultimately advance social justice while simultaneously ensuring the successful acceptability and implementation of an HIV cure.

## Supplementary Material
