# Supplementary material for: Social Engagement in HIV Cure (Research) in the Netherlands: Understanding the Perceived Necessity and Concerns of People With HIV
Source: J Acquir Immune Defic Syndr. 2024 Jun 21;96(4):361–6. doi: 10.1097/QAI.0000000000003429 (PMC11195926; doi:10.1097/QAI.0000000000003429)
Supplement: Supplementary file 1 [file qai-96-361-s001.docx]

**Appendix I - Interview Protocol: Awareness, Importance, and Meaning of HIV Cure and the Role of MIPA, Stigma, and Language in HIV Cure (research)**

Below, you will find a detailed description of the interview instructions. The primary questions are highlighted in **bold**, while optional questions are indicated in *italics and underlined*. The text in *italics* represents the recommended interview script, which can be delivered verbatim or in your own words.

# PREPERATION:

Before the participant arrives, assign a code to the participant; the participant completes the code with two self-chosen digits and enters it into the Qualtrics consent form.

# INTRODUCTION

- - Introduce yourself (the interviewer).
  - Ensure that the participant has read and understands the consent form and that participation is voluntary, with the option to withdraw at any time.
  - Explain the specific goals of the research (Awareness, Importance, and Meaning, MIPA, Stigma, and language usage) and provide an overview of the interview structure: *Today, we will discuss various topics within the field of HIV cure and HIV cure research. We would like to understand your ideas and opinions on these subjects through a series of questions. There are no wrong answers, and it's all about how you perceive and experience these topics. We would also like to emphasize that while we are asking for your ideas and opinions on HIV cure and HIV cure research, this is not a pre-screening for participation in any medical cure studies. The interview will last approximately 1 to 1.5 hours.*
  - Offer the participant the opportunity to ask questions and record the consent.
  - Explain how the interview will be digitally recorded. Start the recorder and read the unique identification code so that the recording can be linked to the consent form.
  - Begin the interview.

# INTRODUCTION QUESTIONS (ONLINE VIA QUALTRICS):

The following information will be collected:

1. Age
2. Gender identity
3. Migration background
4. Sexual identity
5. Living environment (village/city)
6. Province
7. ART usage
8. Years since diagnosis
9. Relationship status

# ROLE OF HIV IN THE PARTICIPANT'S DAILY LIFE

- - **Can you briefly tell us about yourself?**
  - **What significance does HIV hold for you?**
  - **What role does HIV play in your daily life?**

If the participant has difficulty answering the question, one of the following questions can be asked:

- *How has HIV impacted your health?*
- *How do you currently experience your HIV medication (if you are taking medication)?*
- *What influence does HIV have on your daily activities (e.g., work, studies, hobbies)? Do you feel that people react negatively to you because of your HIV status? How do you notice this?*
- *How does HIV affect your sexual behavior?*

# AWARENESS OF HIV CURE (RESEARCH)

- - **What do you know about HIV cure?**

If the participant has difficulty answering the question, one of the following questions can be asked:

- *To what extent is it currently possible to cure HIV?*
- *What do you know about the London patient, the Berlin patient (Timothy Ray Brown), the Mississippi baby?*
  - **What do you know about HIV cure research? Do you follow developments in HIV cure research? If so, how do you follow these developments?**

If the participant has difficulty answering the question, one of the following questions can be asked:

- **What interest do you have in HIV cure research? If you were to start following HIV cure developments, where would you look?**
  - **What information would you like to receive about HIV cure if it becomes a reality?** *From whom would you prefer to receive this information?*
  - **When would you have confidence in a cure for HIV?**

# MEANING OF HIV CURE (RESEARCH)

- - **What would change for you if you no longer had HIV tomorrow?**

If the participant has difficulty answering the question or indicates that their life wouldn't change, the following questions can be asked:

- - *What would change if you no longer had to take medication daily (if you are taking medication)?*
  - *What impact could a cure have on concerns about your health?*
  - *How would a cure affect your daily activities?*
  - *What influence could the possibility of a cure have on the disclosure of your HIV status?*
  - **If you no longer had HIV tomorrow, how do you think people would react?**
  - *How would your partner react (if the person has a partner)? How would your friends/family react?*
  - **How important are these reactions to you?**
  - *How important is the reaction of your partner to you (if the person has a partner)?*
  - *How important are the reactions of your friends/family to you?*
  - **What does an ideal cure for HIV look like to you?**

*Researchers are currently working on two types of HIV cure: one that completely eliminates the virus from the body and another that keeps HIV under control for an extended period without treatment.* **How acceptable are these two different forms of HIV cure to you?**

If the participant finds it difficult to answer these questions, the following questions can be asked:

- - *How acceptable is an HIV cure if there is a chance of the HIV infection returning (e.g., through new infection or the virus becoming active in the body again)?*
  - *What role does the risk of transmitting HIV play in HIV cure?*
  - *How would you feel if you had to regularly visit the hospital for some time to see if your HIV infection is being controlled without treatment?*
  - *In the search for an acceptable HIV cure, various types of research are ongoing.* **What do you understand by HIV cure research?**

If the participant finds it difficult to answer the question above, you can ask the following questions:

- - *What role do non-medical studies play in HIV cure research?*

# IMPORTANCE OF HIV CURE (RESEARCH)

- - **How important do you find HIV cure research?**

If the participant finds it difficult to answer the question above, you can ask the following questions:

- - *Why is it important or not important to conduct research on HIV cure, in your opinion?*
  - *What do you believe are the consequences if no research is conducted on HIV cure? Who benefits from conducting research on HIV cure?*
  - **How important is an HIV cure to you personally, and why?**

If the participant finds it difficult to answer the question above, you can ask the following questions:

- - Do you expect that an HIV cure would bring about a change in your quality of life, and how important is that to you?
  - Do you expect that a cure would improve your health, and how important is that to you?
  - To what extent is it important that a cure can prevent new HIV infections?
  - *HIV cure research may entail risks.* **What risks might be involved in HIV cure research?**

If the participant finds it difficult to answer the question above, you can ask the following questions:

Possible medical risks:

- *What role might potential side effects play?*
- *What role might uncomfortable procedures (such as blood tests, biopsies, or other interventions) play?*
- *How do you view stopping the use of HIV medication during a curative treatment?*
- *How concerned would you be if someone becomes virally detectable during an HIV cure research study?*

Possible social/psychological risks:

- *How would you feel if someone becomes virally detectable again during participation in HIV cure research and could potentially transmit the virus to their (sexual) partner?*
- *What impact might participation in HIV cure research have on your anonymity?*
- *What impact might participation in HIV cure research have on your daily activities?*
- *What impact might participation in HIV cure research have on your relationship with friends and/or family?*
- *When it comes to the risks we've just discussed,* **when are those risks acceptable in HIV cure research?**

If the participant finds it difficult to answer the question above, you can ask the following question:

- *What risks would you personally take to be cured?*

# MEANINGFUL INVOLVEMENT IN HIV CURE RESEARCH

- - **How important do you think it is for people with HIV to be involved in every aspect of research (including designing, conducting, analyzing, interpreting, and disseminating results)?**
    - *To what extent do you believe people with HIV should have influence over HIV cure research, and why do you think so?*
    - *In your opinion, what is the significance of involving people with HIV?*
  - **Would you like to be involved in research? Why or why not?**
    - *How would you prefer to be involved in HIV cure research?*
  - **To your knowledge, what is currently being done to involve people with HIV in HIV cure research?**
  - **How can people with HIV best be engaged in the search for an HIV cure?**If the participant finds it difficult to answer the question, you can ask the following question to help get the conversation going:
  - *How would you like to be involved in HIV cure research? What do you think you can contribute to HIV cure research?*

Regarding the above questions, don't forget to follow up with prompts such as:

- - *What exactly do you mean by that?*
  - *Do you have an example?*
  - *Can you tell me more about it?*
  - *How do you mean?*
  - *Why do you think/feel that way?*

# IMPORTANCE OF HIV CURE (RESEARCH) (STIGMA)

- - *Negative perceptions about people with HIV have existed since the beginning of the HIV epidemic***. To what extent do you think a cure could change these perceptions?**
  - **Could it also change your own perceptions about HIV? How?**

It is important that the participant addresses all different types of stigma (self-stigma, which can be further divided into anticipated stigma [expectations of how others will stigmatize] and internalized stigma [accepting others' stigma as truth], public stigma, and structural stigma [stigma within organizations, culture, and laws]) (see questions below).

- - **Do you think people would view those with HIV differently if it could be cured? In what ways?**
  - **What impact do you think a cure could have on the public's opinion of HIV and people with HIV?**
    - *What changes might a cure for HIV bring in how people with HIV are treated by their surroundings?*
  - **Would you also think differently about yourself and your HIV if there was a cure for HIV? How would that be?**
  - **Do you think a cure for HIV could also lead to new forms of negative perceptions? In what ways?**
    - *Imagine it's possible to contract HIV again after being cured. How would that affect negative perceptions?*
    - *Imagine not everyone can be cured, but, for example, only women or only those under 65 years old. How would that affect negative perceptions***?**
  - **If you could be cured of HIV, would it affect how people in non-HIV-related healthcare treat you? In what way?**

If the participant has difficulty answering, you can ask more specific questions, such as:

- - *How do you think, for instance, your visits to the dentist or other specialists, who are aware of your HIV status, would change when there is a cure for HIV?*
  - *As discussed earlier, researchers are currently working on two types of HIV cure: one where the virus is completely eliminated from the body and another where HIV is kept under control for an extended period without treatment.* **How do you think negative perceptions would differ if the virus were completely eliminated from the body?**
  - **How do you think negative perceptions would differ if the virus were kept under control for an extended period without treatment?**

Regarding the above questions, don't forget to ask for clarification or examples, if necessary:

- - *What exactly do you mean by that?*
  - *Do you have an example? Can you provide more details?*
  - *Why do you think/feel that way?*

# COMMUNICATION & LANGUAGE AROUND HIV CURE AND HIV CURE RESEARCH

- - *Over the years, we have learned that language usage is important. The words we use when talking about HIV matter. Currently, with cure research, we are in relatively new territory.* **What are some things we should pay attention to when discussing/communicating about HIV cure research?**
  - **Do you have ideas about which words we should or should not use when discussing an HIV cure? Which words should we use, and which should we avoid?**

If it doesn't come up explicitly in the participant's response, you can ask the following question:

- - - *Some people believe that 'cure' is not the right term to use; what is your opinion on that?*
    - *It is important to use the correct terminology for different groups. For example, not everyone with HIV sees themselves as a patient and may not want to be referred to as such. Are there other aspects of communication that you find uncomfortable and think should be handled differently?*
  - *We want to optimize the involvement of people with HIV.* **In what way should we discuss HIV cure research to enhance this involvement?**
  - *As we've discussed earlier, negative perceptions are an important topic in the HIV field.* **How should we talk about HIV cure research to ensure that people do not react negatively towards individuals with HIV?**

If further clarification is needed, you can use the following questions:

- - - *Could you please explain why you feel that way?*
    - *Could you provide a few examples?*
    - *What exactly do you mean by that?*
